# Egg shell and yolk quality characteristics of layers fed with sugarcane press residue in soya and fish based diets

**DOI:** 10.14202/vetworld.2015.232-238

**Published:** 2015-02-25

**Authors:** N. Suma, B. S. Venkatarami Reddy, R. G. Gloridoss, T. M. Prabhu, C. Basavanta Kumar, B. N. Suresh, V. T. Shilpa

**Affiliations:** 1 Department of Animal Nutrition, Veterinary College, Karnataka Veterinary, Animal and Fisheries Sciences University, Hassan, Karnataka, India; 2 Department of Animal Nutrition, Veterinary College, Karnataka Veterinary, Animal and Fisheries Sciences University, Bengalure, India; 3 Department of Instructional Livestock Farm Complex, Veterinary College, Karnataka Veterinary, Animal and Fisheries Sciences University, Hassan, Karnataka, India; 4 Department of Veterinary Pathology, Veterinary College, Karnataka Veterinary, Animal and Fisheries Sciences University, Hassan, Karnataka, India

**Keywords:** egg shell thickness, layer, sugarcane press residue, yolk color, yolk index

## Abstract

**Aim:**

Sugarcane press residue (SPR), a by-product of sugarcane industry, which is rich in inorganic salts was assessed at different levels in both soya based and fish based diets of layers for egg shell and yolk quality characteristics.

**Materials and Methods:**

SPR was incorporated in 32-week-old white leghorn layer diets at 0%, 5%, 10% and 15% either in the soya based or fish based diets to form T_1_ to T_8_ diets, respectively. Each diet was offered to five replicates of four laying hens each constituting a total of one sixty birds kept for 84 days under colony cages.

**Results:**

Mean egg shell thickness obtained from eggs of experimental hens measured was 0.342, 0.329, 0.320, 0.322, 0.319, 0.332, 0.328 and 0.336 mm in T_1_ through T_8_ groups, respectively. About the main factor effects, both showed non-significant results. Similarly, influence of different treatment diets, in imparting colour to the yolks, was found to be non-significant (p>0.05) at different 28-day time intervals. Further, the average yolk index values ranged non-significantly from 0.360 (T_6_) to 0.383 (T_4_).

**Conclusion:**

The SPR can be incorporated into layer diet as a source of inorganic as well as organic nutrients without affecting its egg quality characteristics.

## Introduction

Nearly 3% of the crushed sugarcane from sugar industries turns into sugarcane press residue (SPR), which is a valuable source of minerals, as well as organic matter. Layer farming is a well organized sector requiring about 6 m MT of feed annually. As a result, such huge quantity of feed requires the conventional mineral mixture to an extent of 0.18 m MT (i.e. about 3% of feed). Even if 10% of such required mineral mixture is spared by SPR, there would be an annual utilization of 20,000 tons of dried SPR. Recent reports of SPR as an animal feed indicated that, it can be a potential alternative source of inorganic as well as for organic nutrients for livestock and poultry [1-8].

Hence, a study in layers with inclusion of higher levels of SPR was being taken up to assess the egg quality characteristics.

## Materials and Methods

### Ethical approval

This research work was carried out as per the guidelines in force at the time of carrying out the group experiment as well as in accordance with the Institutional Ethics Committee, Veterinary College, Bangalore guidelines to minimize pain or discomfort of the birds. The study was approved by the Institutional Ethics Committee.

### Collection of SPR samples

The SPR for the study was procured from Shivamogga, Karnataka and was dried under sun till it became air dry. The sample was first screened for proximate composition [9] and then for microbiological examination as well as for multimycotoxin estimation. The calcium and phosphorus contents in SPR were analyzed as per the procedure described by [10].

### Formulation of experimental diets

Two BIS specified [11] practical control diets for both soya (T_1_) and fish based (T_5_) test diets were formulated, while the SPR was included at three levels (5%, 10% and 15%) in test diets of both soya based (T_2_-T_4_) and fish based (T_6_-T_8_) types to form a total of 6 treatment diets. Further, enough care was exercised to optimize the levels of most of the essential minerals and Ca:P ratio among various diets.

### Randomization and experimental care

A total of 160 BV-300 commercial layers of about 32 weeks age and uniform body weight were housed in two twin-bird colony cages each measuring 15″ × 15″ × 18″ size to serve as a replication. All birds were divided randomly into 40 groups of 4 birds each, and each of the 8 diets described earlier were offered to five such replications. A completely randomized design was employed to carry out the experiment. The experiment lasted for 84 days, which was conveniently divided into three 28-day interval periods.

### Egg characteristics

All eggs produced in different replicate groups were collected on four occasions, i.e. on 1^st^ day, 28^th^ day, 56^th^ day and on 84^th^ day of the experiment. Each egg so collected during every 28 day intervals was broken open, and the entire contents were carefully placed on a glass slab to analyze different egg characteristics.

### Egg shell thickness

After placing the entire contents of an egg on the glass slab, the shell pieces being made devoid of shell membranes at broad end and a narrow end were carefully selected and their thickness was measured using digital calipers.

### Yolk color

The color of yolk from every broken open egg of different groups at all the 28-day intervals was scored by matching (contrast) technique using Roche yolk color fan [12].

### Yolk index (YI)

YI was calculated for all eggs produced in different groups at every 28-day interval. The yolk height was measured using Ames Haugh Unit Spherometer and diameter by Vernier Calipers. The YI was calculated as:


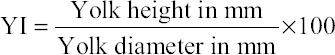


### Statistical analysis

The experimental data were statistically analyzed by two-way ANOVA to separate the factor and interaction effect using GraphPad Prism program [13]. Wherever factor effect was significant (p<0.05), the Bonferroni post-test was used with p<0.05 to compare such means.

## Results and Discussion

Composition of SPR and experimental diets: The chemical composition of the SPR sample (Table-1) revealed that it comprised of crude protein (CP)-12.67, ether extract (EE)-7.50, crude fiber (CF)-17.50, total ash-24.62, nitrogen free extract (NFE)-37.71 and AIA-9.51%. The mineral composition of the said sample of SPR was: Calcium-4.52, Phosphorus-1.25, Magnesium-1.28, Potassium-1.81, Sulphur-2.62%, Iron-2042, Manganese-228.0, Zinc-36.5, Copper-22.6 and cobalt-236.7 ppm.

**Table-1:** Proximate composition and mineral profile of SPR.

Parameter	Level
Proximate composition (%)	
DM	90.77
CP	12.67
EE	7.50
CF	17.50
Total ash	24.62
NFE	37.71
AIA	9.51
Mineral profile (%)	
Phosphorus	1.25
Potassium	1.81
Calcium	4.52
Magnesium	1.28
Sulfur	2.62
Iron (ppm)	2042
Manganese (ppm)	228
Zinc (ppm)	36.5
Copper (ppm)	22.6
Cobalt (ppm)	236.7
Other parameters	
pH	6.35
Organic carbon (%)	40.87

DM=Dry matter, CP=Crude protein, EE=Ether extract, CF=Crude fiber, NFE=Nitrogen free extract, SPR=Sugarcane press residue

The SPR appears to be similar to that of cereal grains in terms of CP (12.67%) and it’s CF content (17.50%) resembles that of brans (De-Oiled Rice Bran); values similar to that reported by Singh and Solomon [14]. Although its total ash of 24.62% and AIA of 9.51% are unique to itself, the highest EE content of SPR (7.50%) might concurrently carry significant quantity of waxes, a rich content of cane sugar. The mineral profile of SPR of the present study is well within the range as reported by [14]. However, these values are slightly different than the composition reported by [15,16]. The variability in composition may be due to quality of the cane crushed, and the process followed for clarification of cane juice in the sugar industry. As expected, from the proximate analysis of layer diets, the contents of crude protein and NFE tended to decline with incremental levels of SPR in such diets. Such a trend was quite opposite for the rest of the nutrients especially for EE and CF.

The SPR samples were also subjected to screening for microbial contamination, which has revealed that they were negative for *Escherichia coli*, *Bacillus* and *Salmonella* species. Further, the sample under study did not carry any mycotoxin with it.

The proximate composition of experimental layer diets compounded on different occasions of the 84-day experimental period is given in Table-2. The results revealed that the proximate analysis of layer diets was similar among all the 8 diets.

**Table-2:** Proximate composition of experimental layer diets (% on DM basis)*.

Dietary description		Treatments	DM	CP	EE	CF	TA	NFE	Ca	P

Protein source	SPR (%)									
Soya based										
Control	0	T1	89.90	17.57	1.91	7.15	13.09	60.28	3.92	0.80
Test	5	T2	89.85	17.36	2.36	7.20	13.25	59.83	3.93	0.76
	10	T3	89.85	17.20	2.91	7.36	14.46	58.07	3.93	0.72
	15	T4	89.37	17.01	3.39	7.42	14.36	57.82	3.93	0.68
Fish based										
Control	0	T5	89.56	17.48	2.26	7.19	15.17	57.91	3.92	0.81
Test	5	T6	89.37	17.39	2.74	7.36	15.37	57.15	3.93	0.77
	10	T7	89.35	17.23	3.30	7.50	15.26	56.71	3.93	0.73
	15	T8	89.26	17.09	3.83	7.60	15.81	55.67	3.93	0.69

* Average values of compounded diets on six occasions, DM=Dry matter, CP=Crude protein, EE=Ether extract, CF=Crude fiber, NFE=Nitrogen free extract, SPR=Sugarcane press residue

### Egg shell thickness

Shell thickness is also an important egg quality factor, which is dependent on dietary regimen among many factors. From Table-3, it can be observed that the dietary groups are statistically (p<0.05) different from each other on 1^st^ day, 56^th^ day and 84^th^ day of the experimental periods. Although values were statistically (p>0.05) similar on 28^th^ day, no definitive trend was observed in any particular dietary treatment. The results further show that the shell thickness generally tended to increase till midway of the experiment but declined at 56^th^ and 84^th^ days of the experiment.

**Table-3:** Average shell thickness (mm) of eggs from experimental birds fed different diets during different time intervals.

Dietary description		Treatments	Average shell thickness (mm)				
	
Protein source	SPR (%)		1^st^ day	28^th^ day	56^th^ day	84^th^ day	Mean
Soya based							
Control	0	T1	0.359^b^±0.008	0.365±0.007	0.335^b^±0.008	0.310^c^±0.006	0.342±0.013
Test	5	T2	0.353^b^±0.006	0.355±0.005	0.326^ab^±0.004	0.283^abc^±0.007	0.329±0.017
	10	T3	0.338^ab^±0.006	0.369±0.007	0.313^ab^±0.008	0.261^a^±0.010	0.320±0.023
	15	T4	0.339^ab^±0.005	0.370±0.015	0.303^a^±0.009	0.276^ab^±0.006	0.322±0.020
Fish based							
Control	0	T5	0.326^a^±0.002	0.364±0.012	0.303^a^±0.005	0.284^abc^±0.004	0.319±0.017
Test	5	T6	0.337^ab^±0.006	0.381±0.006	0.305^a^±0.005	0.306^c^±0.005	0.332±0.018
	10	T7	0.342^ab^±0.006	0.378±0.009	0.298^a^±0.007	0.296^bc^±0.007	0.328±0.020
	15	T8	0.349^ab^±0.007	0.378±0.007	0.306^ab^±0.005	0.311^c^±0.005	0.336±0.017
p value			0.002	0.548	0.077	0.001	0.241

a-c Within a column, means bearing at least one common superscript are statistically similar (p>0.05), SPR=Sugarcane press residue

**Figure-1:**
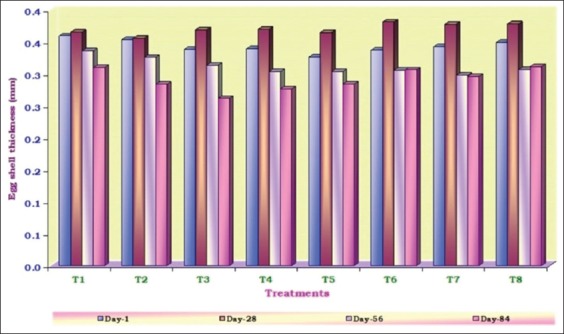
Average shell thickness (mm) of eggs from hens fed different diets at different time intervals.

The average values ranged from 0.326 (T_5_) to 0.359 (T_1_) on 1^st^ day; from 0.355 (T_2_) to 0.381 (T_6_) on 28^th^ day; from 0.298 (T_7_) to 0.335 (T_1_) on 56^th^ day and from 0.261 (T_3_) to 0.311 (T_8_) mm on 84^th^ day (Figure-1). The results of the present study occasionally support the results of [16-19]. The mean egg shell thickness values were 0.331, 0.331, 0.324 and 0.329 mm at 0, 5, 10 and 15 per cent levels of inclusion of SPR. The mean values were almost identical among the protein sources (0.328 and 0.329 mm).

Amongst the main factor effects, the protein source (soya and fish), though inconsistently, did result in significant (p<0.05) differences at all the time intervals except on 1^st^ day. However, SPR levels failed to show any significance during any time interval (Table-4). Although incremental levels of SPR showed inconsistently increased values during initial stages (1^st^ day, 28^th^ day), yet a reduced trend in egg shell strength was observed on 56^th^ and 84^th^ days of the experiment. Egg shell thickness is largely affected by calcium assimilation, under the influence of vitamin D_3,_ including minerals namely zinc and manganese [20]. Inclusion of SPR appears to effectively contribute the said nutrients to support optimal shell thickness since they were replaced to the extent that could be contributable from SPR even at 15% level of inclusion. In fact, the minerals in SPR might exist in the chelated (organic) form as the SPR allows certain microbial fermentation during its procurements and thus allowing better utilization of minerals.

**Table-4:** Average shell thickness (mm) of eggs as affected by main factors at different time intervals.

	Average shell thickness (mm)				
	
	1^st^ day	28^th^ day	56^th^ day	84^th^ day	Mean
i) SPR as main factor					
SPR level (%)					
0	0.342±0.007	0.365±0.007	0.319±0.007	0.297±0.005	0.331±0.015
5	0.345±0.005	0.368±0.006	0.316±0.004	0.295±0.006	0.331±0.016
10	0.340±0.004	0.373±0.005	0.305±0.006	0.279±0.008	0.324±0.021
15	0.344±0.004	0.374±0.008	0.305±0.005	0.294±0.007	0.329±0.018
p value	0.834	0.716	0.073	0.053	0.482
ii) Protein source as main factor					
Protein source					
Soya	0.343±0.003	0.365^a^±0.002	0.316^b^±0.001	0.283^a^±0.002	0.328±0.017
Fish	0.342±0.002	0.379^b^±0.004	0.303^a^±0.003	0.304^b^±0.002	0.329±0.018
p value	0.340	0.011	0.001	0.001	0.890

a-b For a particular main factor, means common superscripts are statistically similar (p>0.05), SPR=Sugarcane press residue

### Yolk color

Egg yolk color is an important quality characteristic from the consumer point of view. The period wise average yolk color scores of eggs of experimental birds fed different diets are presented in Table-5 and the factor wise values being presented in Table-6.

**Table-5:** Average yolk colour scores of eggs from experimental birds fed different diets at different time intervals.

Dietary description		Treatments	Average yolk colour score				
	
Protein source	SPR (%)		1^st^ day	28^th^ day	56^th^ day	84^th^ day	Mean
Soya based							
Control	0	T1	6.43±0.30	6.67±0.18	7.08±0.28	7.33±0.15	6.88±0.20
Test	5	T2	6.73±0.25	6.23±0.18	7.08±0.20	6.98±0.13	6.75±0.19
	10	T3	6.58±0.16	6.76±0.08	7.11±0.12	6.96±0.13	6.85±0.12
	15	T4	6.88±0.19	6.68±0.16	7.09±0.18	7.10±0.07	6.94±0.10
Fish based							
Control	0	T5	6.80±0.05	6.87±0.16	7.09±0.12	7.06±0.06	6.96±0.07
Test	5	T6	6.25±0.19	6.23±0.32	7.13±0.05	7.01±0.06	6.65±0.24
	10	T7	6.44±0.21	6.55±0.21	7.26±0.15	7.00±0.06	6.81±0.19
	15	T8	5.98±0.21	6.77±0.19	7.22±0.13	7.15±0.11	6.78±0.29
p value			0.344	0.745	0.971	0.421	0.752

SPR=Sugarcane press residue

**Table-6:** Average yolk colour scores of eggs as affected by main factors at different time intervals.

	Average yolk colour score				
	
	1^st^ day	28^th^ day	56^th^ day	84^th^ day	Mean
i) SPR as main factor					
SPR level (%)					
0	6.62±0.16	6.77±0.12	7.09±0.15	7.20±0.09	6.92±0.14
5	6.49±0.17	6.23±0.18	7.10±0.10	6.99±0.07	6.70±0.21
10	6.51±0.13	6.65±0.12	7.18±0.10	6.98±0.07	6.83±0.15
15	6.43±0.21	6.72±0.12	7.15±0.12	7.13±0.07	6.86±0.17
p value	0.833	0.081	0.938	0.124	0.312
ii) Protein source as main factor					
Protein source					
Soya	6.68^b^±0.13	6.64±0.09	7.09±0.06	7.09±0.08	6.85±0.14
Fish	6.22^a^±0.10	6.52±0.18	7.20±0.08	7.05±0.06	6.80±0.19
p value	0.046	0.967	0.503	0.677	0.512

a-b For a particular main factor, means common superscripts are statistically similar (p>0.05), SPR=Sugarcane press residue

**Figure-2:**
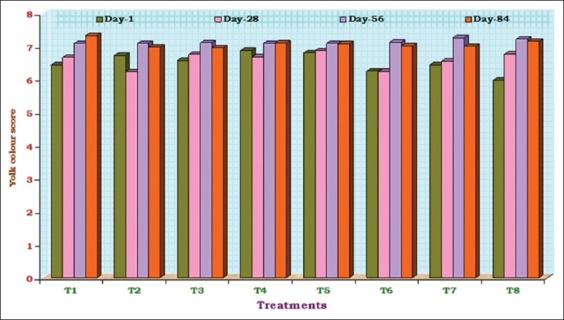
Average yolk colour scores of eggs from experimental birds fed different diets during different time intervals.

The values ranged from 5.98 (T_8_) to 6.88 (T_4_) on 1^st^ day; from 6.23 (T_2_, T_6_) to 6.87 (T_5_) on 28^th^ day; from 7.08 (T_1_, T_2_) to 7.26 (T_7_) on 56^th^ day and from 6.96 (T_3_) to 7.33 (T_1_) on 84^th^ day of the experimental period. The influence of different treatment diets, in imparting color to the yolks, was found to be non-significant (p>0.05) at different 28-day time intervals. The average yolk color scores of eggs measured at different time intervals are graphically presented in Figure-2.

As regards main factors, the yolk color scores (Table-6) were significantly (p<0.05) affected by the protein source factor and that too only on the 1^st^ day with a highest value was 6.68 (soya based) as against lowest value of 6.22 (fish based). In fact such trend might be due to the chance factor as evident by the fact that during successive 28-day intervals, no significant differences could surface between the protein sources. Also observed was the fact that the SPR based diets did not enhance the yolk color intensity which otherwise would have been possible in view of the fact that the SPR *per se* appears to be rich in colouring pigments as that with forages.

### YI

The average YI values of eggs of experimental birds fed different diets during different periods are presented in Table-7 and the main factor wise data are represented in Table-8. From Table-7, it was evident that on 1^st^ day, non-significantly (p>0.05) lower value of 0.346 was noticed in T_6_ as against the highest value of 0.385 observed with T_1_. Contrarily, 28^th^ and 56^th^ day values turned out to be significant (p<0.01) among different treatments. Inclusion of 10%, 5% SPR in fish based diets (T_7_, T_6_) showed significantly lowest values of 0.355, 0.368 as against the highest values of 0.390, 0.401 in 15% SPR included soya based diets (T_4_) during 28^th^, 56^th^ day, respectively. This pattern of significance did not however, persist during the terminal stage (84^th^ day).

**Table-7:** Average YI values of eggs from experimental birds fed different diets during different time intervals.

Dietary description		Treatments	Average YI				
	
Protein source	SPR (%)		1^st^ day	28^th^ day	56^th^ day	84^th^ day	Mean
Soya based							
Control	0	T1	0.385±0.013	0.374^abc^±0.004	0.383^ab^±0.007	0.365±0.003	0.377±0.005
Test	5	T2	0.369±0.013	0.387^bc^±0.008	0.389^ab^±0.007	0.363±0.007	0.377±0.007
	10	T3	0.381±0.005	0.369^abc^±0.007	0.391^ab^±0.005	0.367±0.009	0.377±0.006
	15	T4	0.377±0.006	0.390^c^±0.006	0.401^b^±0.007	0.365±0.011	0.383±0.008
Fish based							
Control	0	T5	0.368±0.004	0.368^abc^±0.005	0.386^ab^±0.006	0.364±0.004	0.372±0.005
Test	5	T6	0.346±0.017	0.358^a^±0.008	0.368^a^±0.001	0.367±0.004	0.360±0.005
	10	T7	0.363±0.006	0.355^a^±0.004	0.369^a^±0.002	0.361±0.009	0.362±0.003
	15	T8	0.364±0.003	0.362^ab^±0.006	0.379^ab^±0.008	0.365±0.005	0.368±0.004
p value			0.975	0.009	0.001	0.918	0.436

a-c Within a column, means bearing at least one common superscript are statistically similar (p>0.05), SPR=Sugarcane press residue, YI=Yolk index

**Table-8:** Average YI values of eggs as affected by main factors at different time intervals.

	Average YI				
	
	1^st^ day	28^th^ day	56^th^ day	84^th^ day	Mean
i) SPR as main factor					
SPR level (%)					
0	0.377±0.007	0.371±0.003	0.384±0.004	0.365±0.365	0.374±0.004
5	0.357±0.011	0.372±0.007	0.379±0.005	0.365±0.365	0.368±0.005
10	0.372±0.005	0.362±0.005	0.380±0.004	0.364±0.364	0.370±0.004
15	0.371±0.004	0.376±0.006	0.390±0.006	0.365±0.365	0.375±0.005
p value	0.249	0.149	0.209	0.999	0.248
ii) Protein source as main factor					
Protein source					
Soya	0.376±0.004	0.378^b^±0.003	0.390^b^±0.004	0.365±0.003	0.378±0.005
Fish	0.358±0.007	0.358^a^±0.002	0.372^a^±0.003	0.365±0.003	0.365±0.004
p value	0.071	0.001	0.001	0.923	0.101

a-b For a particular main factor, means common superscripts are statistically similar (p>0.05), SPR=Sugarcane press residue, YI=Yolk index

The effect observed when the data was analyzed on the basis of main factors was non-significant (p>0.05) as regards the SPR factor was concerned, while the protein source factor revealed significant (p<0.05) differences only on 28^th^ and 56^th^ days of experiment. Inclusion of SPR at 15% showed higher values and also that the soya diets were better than fish diets. Stability of yolk, as reflected by higher YI scores, is important from the point of shelf life of eggs as well as the hatchability. Since SPR contains large amount of lipid portion as wax [16], it might cause mottling of yolks and affect YI, which however did not occur in the present study even at 15% inclusion of SPR. Thus, egg quality is sustainable with SPR even up to 15% in layer diets.

## Conclusion

Egg shell and yolk quality characteristics under the study were not affected by the SPR inclusion in layers. Hence, it can be concluded that, SPR can be incorporated as a non-conventional mineral supplement in layer diet for economic poultry production.

## Authors’ Contributions

NS planned and monitored the work, analyzed the data, drafted the manuscript, BSVR planned, guided and supervised the entire work, reviewed the manuscript. RGG and TMP have given dynamic suggestions during the study. CBK, BNS and VTS have assisted in the work, writing manuscript and reviewed the manuscript. All authors read and approved the final manuscript.
